# Evaluation of the PI-RADS Scoring System for Classifying mpMRI Findings in Men with Suspicion of Prostate Cancer

**DOI:** 10.1155/2013/252939

**Published:** 2013-12-16

**Authors:** Daniel Junker, Georg Schäfer, Michael Edlinger, Christian Kremser, Jasmin Bektic, Wolfgang Horninger, Werner Jaschke, Friedrich Aigner

**Affiliations:** ^1^Department of Radiology, Medical University of Innsbruck, Anichstraße 35, 6020 Innsbruck, Austria; ^2^Department of Pathology, Medical University of Innsbruck, Anichstraße 35, 6020 Innsbruck, Austria; ^3^Department of Urology, Medical University of Innsbruck, Anichstraße 35, 6020 Innsbruck, Austria; ^4^Department of Medical Statistics, Informatics and Health Economics, Medical University of Innsbruck, Schöpfstraße 41/1, 6020 Innsbruck, Austria

## Abstract

*Purpose*. To evaluate the ESUR scoring system (PI-RADS) for multiparametric MRI of the prostate in clinical routine and to define a reliable way to generate an overall PI-RADS score. *Methods*. Retrospective analysis of all patients with a history of negative prebiopsies, who underwent 3 Tesla multiparametric MRI from October 2011 to April 2013 (*n* = 143): PI-RADS scores for each single modality were defined. To generate the overall PI-RADS score, an algorithm based approach summing up each single-modality score to a sum-score was compared to a more subjective approach, weighting the single modalities dependent on the radiologist's impression. Because of ongoing cancer suspicion 73 patients underwent targeted mpMRI-ultrasound image fusion rebiopsy. For this group thresholds for tumor incidences and malignancy were calculated. *Results*. 39 (53%) out of 73 targeted rebiopsies were cancer positive. The PI-RADS score correlated well with tumor incidence (AUC of 0.86, 95% CI 0.78 to 0.94) and malignancy (AUC 0.84, 95% CI 0.68 to 0.99). Regarding the sum-score a threshold of ≥10 turned out to be reliable for cancer detection (sensitivity 90%, specificity 62%) and for ≥13 for indicating higher malignancy (Gleason ≥4 + 3) (sensitivity 80%, specificity 86%). To generate the overall PI-RADS score, the use of an algorithm based approach was more reliable than that of the approach based on the radiologist's impression. *Conclusion*. The presented scoring system correlates well with tumor incidence and malignancy. To generate the overall PI-RADS score, it seems to be advisable to use an algorithm based instead of a subjective approach.

## 1. Introduction

Due to the increasing availability of multiparametric magnetic resonance imaging (mpMRI) in general, the improved image quality at 3 Tesla, and the increasing number of studies confirming the diagnostic reliability of mpMRI for prostate cancer (PCa) detection, mpMRI proceeds to become an important and widely used tool for PCa diagnosis [1–5]. In Austria we recognize a growing urological demand for mpMRI of the prostate especially in patients with negative systematic prebiopsies but ongoing tumor suspicion.

The multiparametric approach using three different MRI techniques (T2-weighted MRI (T2W-MRI), diffusion-weighted imaging (DWI), and dynamic contrast-enhanced MRI (DCE-MRI)) can improve the diagnostic accuracy. However its complexity and the sometimes contradictory findings of the different single modalities may result in a wide scope of possible interpretations of mpMRI findings leading to heterogeneities between different readers and different diagnostic centers [6–9].

To overcome these problems, the European Society of Urogenital Radiology (ESUR) has called a panel of experts and published a guideline providing recommendations for the performance of mpMRI investigations and a structured reporting scheme named Prostate Imaging Reporting and Data System (PI-RADS) in February 2012 [10]. Inspired by the BI-RADS system for breast cancer detection [11, 12], this scheme is based on Likert scales, scoring each single modality from 1 to 5 (single-scores) and then generating the final PI-RADS score (1–5 points: overall PI-RADS). Similar to BI-RADS, the PI-RADS score involves individual risk stratification for the absence or presence of a clinically relevant disease and should be part of the written report. Although the ESUR guideline provides explicit criteria for how to generate each single-score, it lacks a consistent instruction on how to calculate the overall PI-RADS score [10, 13, 14].

The aim of this study was to evaluate the PI-RADS scoring system in our patient population and to find the best way of generating the overall PI-RADS score.

## 2. Materials and Methods

### 2.1. Patients

From October 2011 to May 2013, 143 consecutive patients with a history of at least one negative systematic prebiopsy, who underwent 3 Tesla mpMRI of the prostate because of ongoing tumor suspicion, were included in this retrospective single-center study in Innsbruck. None of the patients were under treatment with 5-alpha-reductase inhibitors at the time the MRI was performed. Of these 143 patients, 73 underwent systematic and targeted rebiopsy within 3 months after mpMRI. Patient characteristics are summarized in Table 1. A positive vote of the local ethical committee was obtained.

### 2.2. mpMRI Technique

mpMRI was performed on a 3 Tesla whole body scanner (Magnetom Skyra, Siemens AG, Erlangen, Germany) using an 18-channel phased array body coil with 18 integrated preamplifiers. Examinations included 2D and 3D T2W-MRI, DWI, and DCE-MRI. MRI parameters are shown in Table 2. 2D T2W-MR images were obtained in axial orientation using T2W turbo spin echo (TSE) sequences covering the entire prostate and the seminal vesicles. For 3D T2W images a 3D TSE sequence with variable flip angle (3D SPACE sequence) was used in sagittal orientation. DWI was obtained in axial orientation using a spin echo-echo planar imaging (SE-EPI) sequence with three *b*-values (50, 400, and 1000 s/mm²) and restriction of diffusion was quantified by the apparent diffusion coefficient (ADC) value [15, 16]. DCE-MR images were obtained using fast 3D T1-weighted (T1-VIBE) gradient echo sequence in axial orientation every 7 seconds for about 10 minutes. As a contrast agent Gadobutrol (Gadovist, Bayer Schering Pharma, Germany) was used with a dose of 0.1 mL/kg body weight. Bolus injection was performed using a power injector (3T Tennessee, Ulrich, Germany) with a flow rate of 0.1 mL/s. Perfusion curves were generated with the commercial software TISSUE4D (Siemens AG, Erlangen, Germany) [17] which was available on the MR scanner console.

#### 2.2.1. Image Interpretation

The mpMRI datasets were analyzed by two experienced uroradiologists with at least 6 years of experience in prostate MRI interpretation, who compared two different approaches to generate an overall PI-RADS score.

In a first step the three single-scores (1–5) for T2W-MRI, DWI, and DCE-MRI for each patient were defined according to the ESUR guidelines (Table 3) by the two radiologists in consensus.

Subsequently, in a first algorithm based approach the first radiologist calculated a PI-RADS sum-score (scale from 3 to 15) by summation of the 3 single-scores. The overall PI-RADS score (1–5) was obtained by classifying the sum-score according to the algorithm proposed by Röthke et al. [13] (Table 4, column 3).

In a second more subjective approach the second radiologist independently generated an overall PI-RADS score by subjectively weighting the results of the single-scores according to the definitions of the ESUR panel (Table 4, column 2), but without deriving it from a strict algorithm. So whenever the results of the three single-scores were incoherent, the analyzing radiologist had to prefer one of the single modalities over the others.

Image interpretation and scoring were done before biopsy, so the radiologists were blinded to the histopathological outcomes. For reporting and localization of findings previous to targeted biopsies the prostate was divided into 27 regions as recommended by the ESUR guidelines according to a scheme presented by Röthke et al. [10, 13].

#### 2.2.2. Rebiopsy

**Figure 1:**
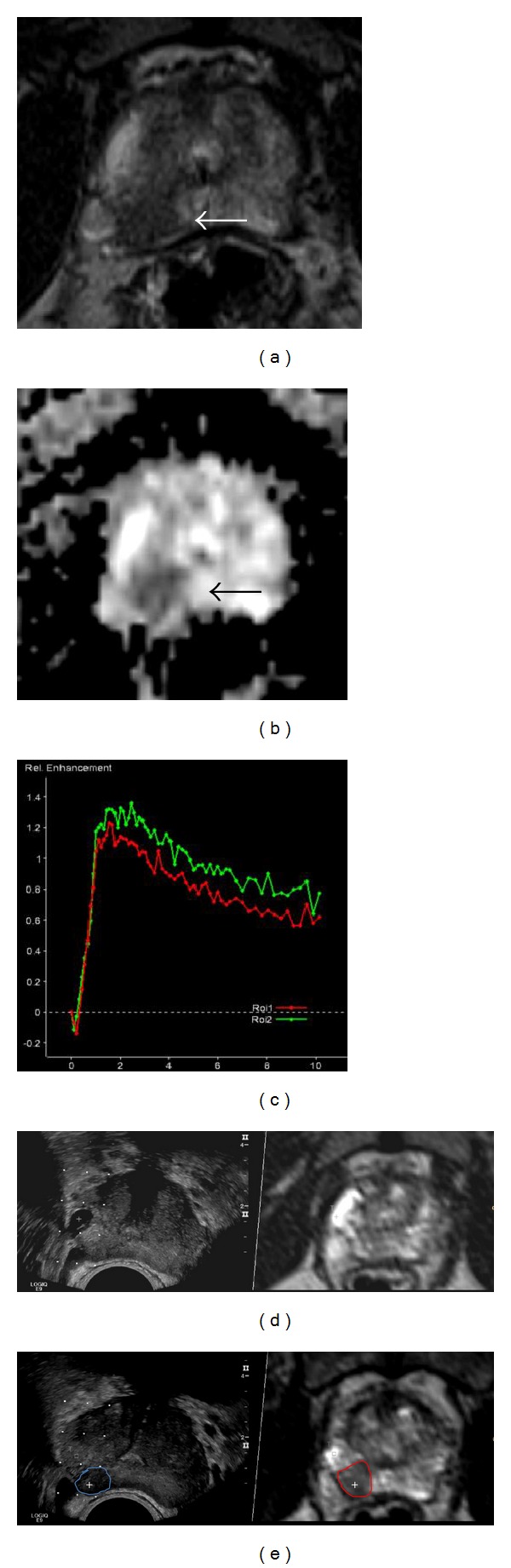
mpMRI-ultrasound image fusion: suspicious lesion (arrows) on T2W (a), on DWI with low ADC (b), and washout curve on DCE (c). Correlation of an anatomical landmark (cyst) for registration of *b*-mode ultrasound and SPACE 3D T2W-MRI (d). Target point (+) in the center of the suspicious lesion on *b*-mode ultrasound and SPACE 3D T2W-MRI (e). Note the slight deformation of the lesion (circle) on the ultrasound due to compression by the endorectal probe.

Within 3 months after mpMRI 73 patients underwent re-biopsy, which was indicated in consideration of radiological and clinical findings by the attending urologist. Within one re-biopsy setting one of the uroradiologists, who interpreted the mpMRI images, took 5 targeted cores of those lesions that were suspicious on at least one single modality (PI-RADS sum-score ≥7). Additionally the urologist, who was unaware of the mpMRI imaging results, took 10 systematic cores of all patients. All rebiopsies were taken with an ultrasound system equipped with an endfire endorectal biopsy probe (Logiq 9 ultrasound unit, GE Healthcare, Little Chalfont, United Kingdom, or EUB 8500 Hitachi ultrasound unit, Hitachi Medical Systems, Tokyo, Japan). Targeted re-biopsy was performed with mpMRI-ultrasound fusion. Registration of suspicious lesions was done with a combined approach of cognitive evaluation on the basis of zonal anatomy and imaging landmarks as well as computerized real-time 3D transrectal US-MRI image fusion by uploading the SPACE 3D T2W sequence to the Logiq 9 ultrasound system [18–20] (Figure 1).

For histopathological analysis all biopsy specimens were numbered, reviewed by a pathologist with >10 years of experience in prostate characterization, and reported as PCa with an assigned Gleason score, prostatitis, adenomyomatosis, benign prostatic hyperplasia, or atrophy.

### 2.3. Statistical Analysis

Summary statistics are provided using the appropriate measures of location and measures of variation for all 143 patients. The D'Agostino-Pearson test was used to test for normal distribution. Mean values ± standard deviations were given for normal distributed data and otherwise median with interquartile range. The different approaches to generate the overall PI-RADS score were compared regarding number and distribution of score levels for all patients within the collective.

Correlation of mpMRI findings and histopathological findings was performed only for the collective of 73 patients, who underwent re-biopsy: to assess a possible positive association between the number of biopsies conducted before the re-biopsy and the relative number of tumor cases, a Chi-squared test for trend was applied. A receiver operating characteristic (ROC) analysis was performed to evaluate sensitivity and specificity of the scoring system with regard to tumor incidence and tumor malignancy. For statistical analysis respective to tumor malignancy, histopathologic results were split into two groups (Gleason score level ≤3 + 4 versus Gleason score level ≥4 + 3). Additionally, an assessment of cutoff levels was made. Two-sided *P* < 0.05 was considered statistically significant. The statistical calculations were performed using SPSS 19.0.

## 3. Results

### 3.1. Biopsy Results

After performing mpMRI, 39 (53%) out of 73 targeted rebiopsies were positive for prostate cancer. Of the 39 tumors, 22 (56%) were located in anterior parts of the prostate, and 17 (44%) in the transitional zone (TZ) while 17 tumors (44%) were located in the posterior parts and 22 (56%) in the peripheral zone (PZ). Regarding tumor malignancy, 29 (74%) were cancers with Gleason ≤3 + 4 and 10 (26%) cancers with Gleason ≥4 + 3. Chi-squared trend analysis revealed a significant association between increasing tumor incidence and increasing number of negative prebiopsies (*P* < 0.05). Targeted biopsies of suspicious lesions revealed markedly more negative findings within the TZ (83%) than in the PZ (17%) and were caused by the presence of adenomas (58%) or inflammations (42%).

### 3.2. Evaluation of the PI-RADS Single- and Sum-Scores

**Figure 2:**
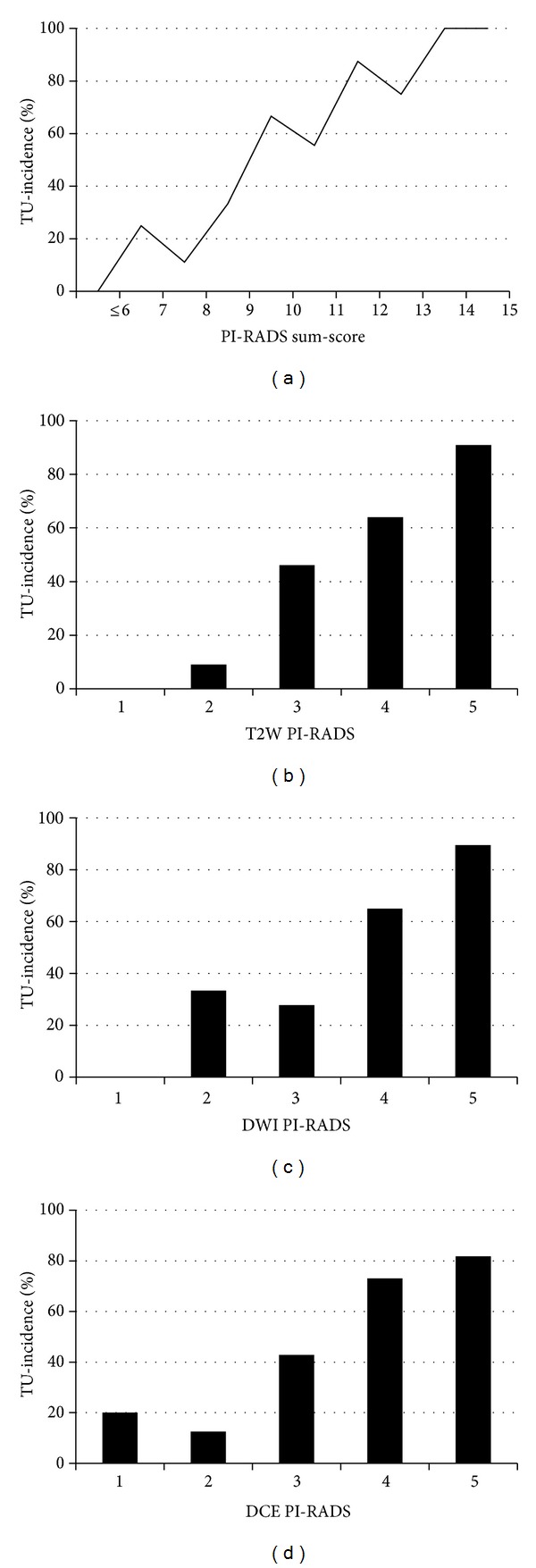
Distribution of tumor incidences for PI-RADS single-scores and sum-scores.

**Figure 3:**
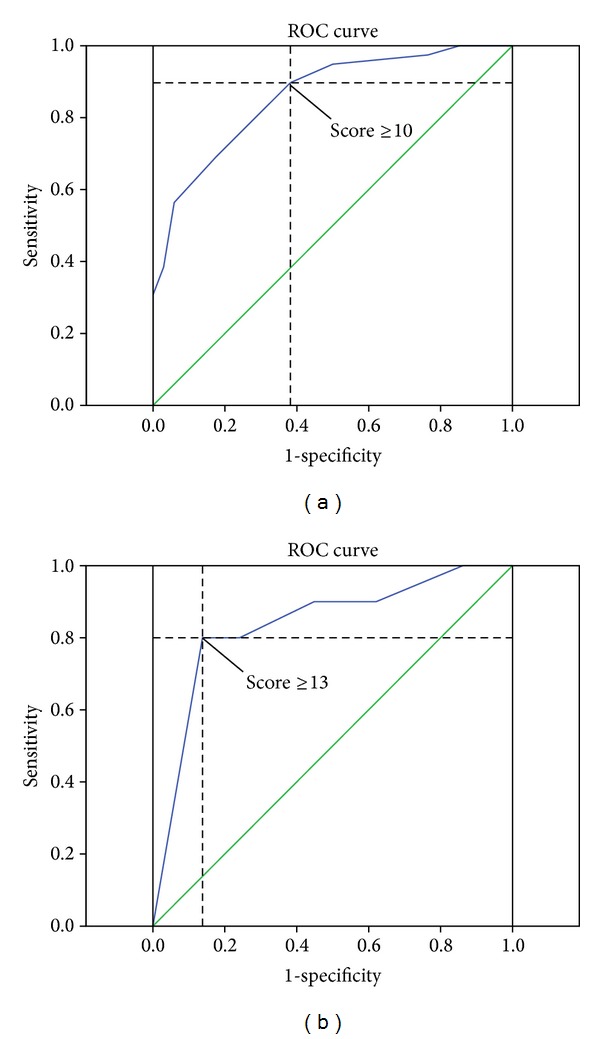
Receiver operation characteristic (ROC) curves for the PI-RADS sum-score, regarding thresholds for tumor incidence with a cutoff at 10 (a) and for tumor malignancy with a cutoff at 13 (b).

**Figure 4:**
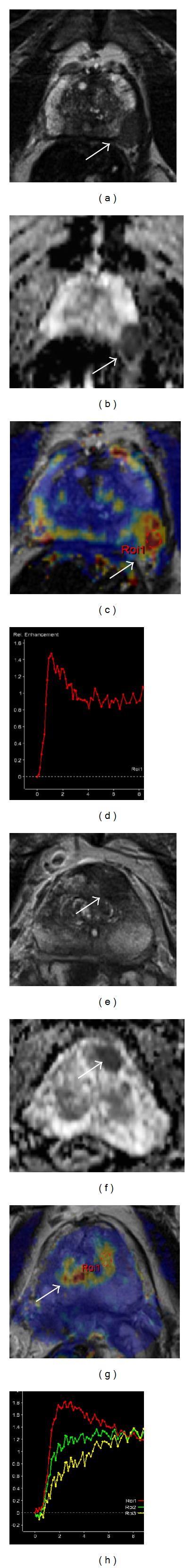
Suspicious lesions (arrows) on mpMRI with different PI-RADS sum-scores. Gleason 8 carcinoma: 5 points on T2W for hypointensity and bulging (a), 5 points on DWI for focal very low ADC (b), and 5 points on DCE-MRI for washout curve in a focal lesion (c, d) = sum-score of 15 points. Gleason 7 (3 + 4) carcinoma with 4 points on T2W for focal hypointensity (e), 5 points on DWI for focal very low ADC (f), and 3 points on DCE-MRI for symmetrical washout curve without focal lesion (g, h) = sum-score of 12 points.

After evaluating the 3 single modalities and adding the single-scores, the collective of 143 patients revealed sum-scores with a median of 8 (range 4–15, IQR 6 to 10). In the group of patients with targeted re-biopsy the PI-RADS sum-score was positively related to the number of cancer positive cores (*P* < 0.05). Each of the single-scores generally showed a tendency to a higher tumor incidence at higher score levels (Figure 2). The ROC analyses revealed a rather large area under the curve (AUC) of 0.86 (95% CI 0.78 to 0.94) regarding tumor incidence and 0.84 (95% CI 0.68 to 0.99) regarding tumor malignancy (Figure 3). When analyzing the balance between sensitivity and specificity to calculate a reliable threshold for tumor incidence for the PI-RADS sum-score, the score level of ≥10 with an accent on sensitivity (90%) rather than specificity (62%) was the highest possible threshold with more sensitivity than specificity. The threshold of ≥11 already showed a markedly lower sensitivity (69%), but better specificity (82%). Tumor incidences differed significantly for score levels below both thresholds compared to those above (*P* < 0.005). Regarding tumor malignancy a threshold was calculated for a score level of ≥13, which revealed high sensitivity (80%) and specificity (86%) for the prediction of cancers with Gleason score ≥4+3. The number of cancers with high Gleason scores (≥4 + 3) differed significantly for score levels below this threshold compared to those above (*P* < 0.005) (Figure 4).

### 3.3. Comparison of Two Different Approaches to Generate the Overall PI-RADS Score (Table 5)

Both, the first approach based on the algorithm of Röthke et al. (PI-RADS scheme 1) and the second approach (PI-RADS scheme 2), based on the overall impression of the radiologist, revealed overall PI-RADS scores, which showed increasing tumor incidence with increasing score levels. When classified according to the algorithm of Röthke et al., it is noticeable that their cutoff between overall PI-RADS 3 and 4 corresponds to the calculated threshold for tumor incidence on the PI-RADS sum-score and their cutoff between 4 and 5 to our calculated threshold for higher tumor malignancy. According to this approach, the prostates of 47 (33%) patients revealed cancer suspicious lesions (PI-RADS scores of either 4 or 5) of which 35 (82%) proved to be cancer positive after targeted biopsy. When generating the overall PI-RADS score simply by the radiologist's impression on the other hand 55 (38%) prostates revealed cancer suspicious lesions, but only 37 (67%) of these proved to be cancer positive after targeted biopsy. Regarding the frequency of PI-RADS 3 lesions, both approaches assigned a similar number of patients to this score level. Nevertheless with 19% compared to 17% biopsy proved tumor incidence in PI-RADS 3 patients was slightly higher for PI-RADS scheme 1. PI-RADS 1 and 2, which mean low suspicion for clinically relevant disease, were diagnosed in 44 (31%) patients when using PI-RADS scheme 1 and in only 38 (27%) patients with PI-RADS scheme 2. None of the biopsies taken from these patients revealed cancer positive cores. The very rare diagnosis of PI-RADS 1 in both approaches can be explained by the presence of multiple tissue alterations in this collective of patients with negative prebiopsies (Table 5).

## 4. Discussion

With this study we could demonstrate a good reliability of the PI-RADS risk stratification system for the interpretation of mpMRI in our patient population: all 3 single-scores and thus the calculated PI-RADS sum-score of 3–15 points showed a clear association with tumor incidence and tumor malignancy with large AUC in ROC curve analysis. In concordance with the other studies, which recently evaluated the PI-RADS classification system with slightly different approaches, this suggests high reliability for the use of a system with fixed criteria for mpMRI interpretation [14, 21, 22]. Similar to the findings of Portalez et al. the T2W single-score proportionally increased with tumor incidence [22]. However, DWI and DCE-MRI single-scores showed indentations at score levels 2 and 3. For DCE-MRI this was mainly due to the observation of several symmetrical or asymmetrical plateau curves in TZ regions, which consequently received 2 points for the DCE-MRI single-score but still were cancer negative after targeted biopsy. This finding mainly corresponded to the presence of adenomas. Low cancer incidences at DWI score level 3 could probably be explained by the existence of fibrous tissue and inflammation in nearly all prostates after systematic prebiopsies, which lead to a certain extent of asymmetrical diffusion restriction and thus were scored with 3 points. All tumors that were found in single-score levels <4 on T2W-MRI, DWI, or DCE-MRI were only carcinomas ≤ Gleason 3 + 4.

Regarding sensitivity and specificity levels of the PI-RADS sum-score on ROC analysis, our results suggest either ≥10 or ≥11 as possible thresholds for the increase of tumor incidence. The question of which of these two values should be used as a threshold to indicate distinct cancer suspicion was discussed with our clinicians, who clearly favored the threshold of ≥10 points for its very high sensitivity level of 90% with an acceptable specificity level of 62%. This goes along to the findings of Schimmöller et al., who also evaluated the sum-score level of ≥10 to be the threshold for tumor incidence and reported a sensitivity of 85.7% and a specificity of 67.6% [21]. However there are some differences when compared to the findings of Portalez et al., who proposed a threshold of ≥9, because of an overall lower sensitivity. This can be explained by the different approaches of our studies, as the main goal of the study of Portalez et al. was to compare targeted biopsy cores with systematically taken cores, and thus they took the single biopsy core as the smallest comparable unit for statistical analysis [22]. We on the other hand wanted to evaluate the PI-RADS scale for its reliability as a risk stratification system for the patient and thus we compared mpMRI findings to the findings of the complete targeted biopsy set (at least 2 cores) in the style of other studies, which dealt with mpMRI/US fusion targeted biopsies [18, 23].

The second goal of this study was to find a reliable approach to generate the overall PI-RADS score, which in the end shall be part of the clinical report as a simplified risk stratification system and which could provide recommendations for further diagnostic procedures. Regarding this issue, the ESUR guidelines lack a consistent instruction of how to generate the overall PI-RADS score [10]. Therefore Röthke et al. published a suggestion to flesh out the ESUR guidelines. According to this proposal the single modalities are added up to a sum-score, which then is classified according to a separate algorithm [13]. However, the authors noted that no evidence-based data exist for certain thresholds (≥10 and ≥13). Rosenkrantz et al. on the other hand presented a study, where the sum-score was not separately classified but interpreted for itself, and additionally an overall PI-RADS score (1–5) was derived from an overall impression by the radiologist according to the definitions provided by the ESUR panel [10, 14].

Comparing the algorithm of Röthke et al. [13] it turns out that their cutoffs between overall PI-RADS 3 and 4 and between 4 and 5 are exactly consistent with our calculated thresholds for tumor incidence (sum-score ≥10) and tumor malignancy (sum-score ≥13), and thus their approach to classify the overall PI-RADS from the sumscore seems to be reliable in accordance with our data.

The second approach to generate an overall PI-RADS score, based on the radiologist's impression, showed less association with the thresholds of the sum-score, and the evaluating radiologist assigned more prostates to PI-RADS 4 and 5, which lead to lower tumor incidences (67% compared to 82%) in these categories and thus less specificity. Regarding PI-RADS 4 and 5 as possible indications for re-biopsy this would have led to a higher number of interventions with a higher percentage of negative results. Therefore, according to our data, an algorithm based approach, which derives the overall PI-RADS score from the sum-score seems to be more reliable.

However, the overall PI-RADS score, recommended by Röthke et al. [13], also led to a noticeable amount of PI-RADS 3 (36%) scores and at the same time showed low cancer incidences in this group (19%). Keeping in mind that PI-RADS 3 is defined as equivocal cancer suspicion and compared to the BI-RADS scoring system of the breast could lead to certain management challenges [24]. To reduce the number of PI-RADS 3, without substantially reducing specificity, we recommend lifting the threshold between PI-RADS 2 and 3 from sum-score levels ≥7 to ≥8. Applied to our patient collective 16 mpMRIs would be reduced from PI-RADS 3 to PI-RADS 2 and thus the rate of PI-RADS 3 scores would be reduced from 36% to 25%. With this correction one Gleason 6 (3 + 3) tumor would have been assigned to score level PI-RADS 2 elevating the tumor incidence to 11% (Table 6).

This study is prone to some limitations. This study was designed as an evaluation of our clinical routine and not every patient underwent re-biopsy of the prostate. This might have led to a verification bias, since patients with few or no abnormalities on mpMRI less frequently underwent re-biopsy. Furthermore in patients without suspicious lesion on at least one single modality (sum-score <7) no targeted biopsies could be performed and systematic re-biopsy had to be used as a gold standard. Therefore all tumor incidences, calculated for low PI-RADS score levels (sum-score <7 or overall Pi-RADS 1 and 2), should be regarded as uncertain. Further studies with data based on a long followup will be necessary to evaluate reliable tumor incidences for these low suspicion groups. Additionally, since each of the evaluating radiologists used a different approach for scoring, we do not have data about interobserver variability within the same approach. For this we refer to a recent study of Schimmöller et al. [21].

## 5. Conclusion

The PI-RADS sum-score (3–15) shows a strong relation to tumor incidence and malignancy in our routine setting for PCa diagnosis. A score level of ≥10 seems to be an important threshold for a positive tumor diagnosis and of ≥13 for the existence of high Gleason scores (≥ 4+3). For generating the overall PI-RADS score, which is part of the clinical report, our results indicate a recommendation for a number based algorithm with a slightly elevated threshold between PI-RADS 2 and 3 compared to that of Röthke et al. [13].

## Figures and Tables

**Table 1:** Patient characteristics at the date of mpMRI.

	All patients (*n* = 143)	Patients with rebiopsy (*n* = 73)
Age (years), mean (s.d.)	62 (7.8)	62 (7.4)
Prostate volume (cm³), median (interquartile range)	45 (34–60)	45 (34 to 61)
Negative prebiopsies, *n* (%)		
1	51 (36%)	17 (23%)
2	50 (35%)	31 (42%)
3	23 (16%)	15 (21%)
4	13 (9%)	6 (8%)
≥5	6 (4%)	4 (5%)
PSA (ng/mL), median (IQR)	6.4 (5.0–11.3)	7.0 (5.1 to 12.9)
Free PSA (%), median (IQR)	13.8 % (11.0%–18.45%)	13.4% (10%–18.6%)

**Table 2:** mpMRI parameters.

	T2W-MRI	DWI	DCE-MRI
Sequence	Fast spin echo	Spin echo EPI	T1w-3D FLASH
TR (ms)	4891	6800	2.89
TE (ms)	101	67	1.12
Flip angle (°)	160	90	2
FoV (mm²)	200*×*200	210 × 210	380 × 285
Resolution	320*×*320	160*×*132	256 × 192
Slice thickness (mm)	3	3	4
*b*-values	—	50/400/1000	—

TR: relaxation time, TE: emission time, FoV: field of view, and FLASH: fast low angle shot magnetic resonance imaging.

**Table 3:** Single-modality scores according to the ESUR panel [10].

	*(A1) T2W imaging for the peripheral zone *
	(1) Uniform high signal intensity
	(2) Linear, wedge-shaped, or geographical areas of lower signal intensity, usually not well demarcated
	(3) Intermediate appearances not in categories 1/2 or 4/5
	(4) Discrete, homogeneous low-signal focus/mass confined to the prostate
	(5) Discrete, homogeneous low-signal-intensity focus with extracapsular extension/invasive behavior or mass effect on the capsule (bulging) or broad (>1.5 cm) contact with the surface
	*(A2) T2W imaging for the transition zone *
	(1) Heterogeneous transition zone adenoma with well-defined margins: “organized chaos”
	(2) Areas of more homogeneous low signal intensity, however, well marginated, originating from the transition zone/benign prostatic hyperplasia
	(3) Intermediate appearances not in categories 1/2 or 4/5
	(4) Areas of more homogeneous low signal intensity, ill defined: “erased charcoal sign”
	(5) Same as 4, but involving the anterior fibromuscular stroma or the anterior horn of the peripheral zone, usually lenticular or water-drop shaped
	*(B) Diffusion-weighted imaging *
	(1) No reduction in ADC compared with normal glandular tissue; no increase in signal intensity on any high-*b*-value image (≥*b*800)
	(2) Diffuse, hyper signal intensity on ≥*b*800 image with low ADC; no focal features; however, linear, triangular, or geographical features are allowed
	(3) Intermediate appearances not in categories 1/2 or 4/5
	(4) Focal area(s) of reduced ADC but isointense signal intensity on high-*b*-value images (≥*b*800)
	(5) Focal area/mass of hyper signal intensity on the high-*b*-value images (≥*b*800) with reduced ADC
	*(C) Dynamic contrast-enhanced MRI *
	(1) Type 1 enhancement curve
	(2) Type 2 enhancement curve
	(3) Type 3 enhancement curve
	(+1) For focal enhancing lesion with curve types 2-3
	(+1) For asymmetric lesion or lesion at an unusual place with curve types 2-3

**Table 4:** Calculation of the overall PI-RADS score according to the definitions of the ESUR panel compared to the algorithm presented by Röthke et al. [13].

Overall PI-RADS	Definition of the ESUR panel	Sum-score of T2W, DWI, and DCE
Score 1	Clinically significant disease highly unlikely to be present	3, 4
Score 2	Clinically significant cancer is unlikely to be present	5, 6
Score 3	Clinically significant cancer is equivocal	7–9
Score 4	Clinically significant cancer is likely to be present	10–12
Score 5	Clinically significant cancer is highly likely to be present	13–15

**Table 5:** Overall PI-RADS score according to Röthke et al. [13] (calculation based on sum-score results) compared to the one based on the overall impression of the radiologist.

Overall PI-RADS score level (1–5)	Score based on Röthke et al.		Score based on radiologist's impression	
	Frequency of patients *n* (%)	Tumor incidence (% of biopsies)	Frequency of patients *n* (%)	Tumor incidence (% of biopsies)
1	1 (1%)	—	0 (<1%)	—
2	43 (30%)	0%	38 (27%)	0%
3	52 (36%)	19%	50 (35%)	17%
4	31 (22%)	65%	38 (27%)	54%
5	16 (11%)	94%	17 (12%)	100%

**Table 6:** Recommendation to calculate an overall PI-RADS score, based on division from the sum-score, with tumor incidences derived from our data.

Overall PI-RADS score	Sum-score of T2W, DWI, and DCE-MRI	Number of patients (%)	Tumor incidence (% of biopsies)	Definition of the ESUR panel
1	3, 4	1 (1%)	—	Clinically significant disease highly unlikely to be present
2	5, 6, 7	59 (41%)	11%	Clinically significant cancer unlikely to be present
3	8-9	36 (25%)	19%	Clinically significant cancer is equivocal
4	10–12	31 (22%)	65%	Clinically significant cancer likely to be present
5	13–15	16 (11%)	94%	Clinically significant cancer highly likely to be present

Changes in comparison to the system of Röthke et al. [13] are underlined (threshold between PI-RADS 2 and 3).
